# Emerging roles of stress granules in liver diseases: a comprehensive review

**DOI:** 10.3389/fcell.2025.1731227

**Published:** 2025-12-04

**Authors:** Qianyun Zhang, Yun Liu, Junzhe Tang, Ming Li, Qiang Jia

**Affiliations:** 1 Shandong Academy of Occupational Health and Occupational Medicine, Shandong First Medical University and Shandong Academy of Medical Sciences, Jinan, Shandong, China; 2 Hospital-Acquired Infection Control Department, Qingdao Traditional Chinese Medicine Hospital, Qingdao Hiser Hospital Affiliated of Qingdao University, Qingdao, Shandong, China; 3 School of Public Health, Shandong First Medical University and Shandong Academy of Medical Sciences, Jinan, Shandong, China

**Keywords:** stress granules, hepatocellular carcinoma, viral hepatitis, acute liver injury, fatty liver disease

## Abstract

Stress granules (SGs) are transient, membraneless condensates that assemble dynamically within cells in response to diverse stressors. In recent years, SGs have been found to be closely associated with multiple pathological states and have attracted significant attention, particularly concerning their roles in hepatic pathophysiology. Functioning as critical hubs for post-transcriptional regulation, SGs maintain cellular homeostasis through the sequestration, transport, and translational suppression of mRNA, thereby potentially modulating the initiation and progression of various liver diseases. Our review summarizes the assembly mechanisms of SGs and recent research advances concerning their involvement in diseases including hepatocellular carcinoma, viral hepatitis, acute liver injury and fatty liver disease. It particularly focuses on SGs core RNA-binding proteins and associated regulatory networks. Although research into the impact of SGs on liver diseases remains in a nascent phase, with mechanistic details still elusive, SGs emerge as pivotal molecular nexuses connecting cellular stress responses to pathophysiological states, highlighting their therapeutic potential for liver disorders. This review aims to provide a theoretical foundation for a deeper understanding of the roles of SGs in liver pathologies and to promote their further development in both fundamental research and clinical translation.

## 1 Introduction

Stress Granules (SGs) are membrane-free ribonucleoprotein (RNP) organelles that form in the cytoplasm of cells under various stress conditions, such as heat shock, oxidative stress, viral infection, osmotic stress, UV irradiation and cold shock. These granules typically range in size from 100 to 2000 nm (Hofmann et al., 2021). In the 1980s, structures similar to SGs were observed in both plant and animal cells. In 1983, Nover et al. reported that tomato cells formed cytoplasmic heat shock granules (HSGs) under heat shock conditions, marking one of the earliest observations of SGs-like structures (Nover et al., 1989). In 1986, Collier and Schlesinger documented dynamic changes in heat shock proteins in chicken embryo fibroblasts and identified similar cytoplasmic granules (Collier and Schlesinger, 1986). As research advanced, a more detailed understanding of the composition and architecture of SGs emerged (Anderson and Kedersha, 2002). SGs are now known to primarily consist of stalled translational messenger ribonucleoproteins (mRNPs) in mammalian cell (Protter and Parker, 2016). Among these, RNA-binding proteins (RBPs), such as T-cell intracellular antigen-1 (TIA-1), TIA-1-related protein (TIAR) (Kedersha et al., 1999; Kedersha et al., 2000) and Ras-GTPase-activating protein SH3 domain-binding protein 1/2 (G3BP1/2) (Matsuki et al., 2013), have been identified as key components. Mechanistic studies have revealed that the phosphorylation of eukaryotic initiation factor 2 alpha (eIF2α) plays a central role in SGs assembly (Kedersha et al., 1999). These early discoveries laid the groundwork for subsequent in-depth investigations into the biology, composition and functional significance of SGs. The liver, as a vital organ responsible for metabolism and detoxification, is highly susceptible to diverse pathological insults including viral infection, oxidative stress, and metabolic dysregulation. Accumulating evidence indicates that SGs are extensively implicated in the progression of various liver diseases. Therefore, this review focuses on SGs assembly mechanisms and summarizes their pathophysiological roles across different types of liver diseases, thereby serving as a comprehensive reference for future research.

## 2 Processes of stress granules assembly and disassembly

**FIGURE 1:**
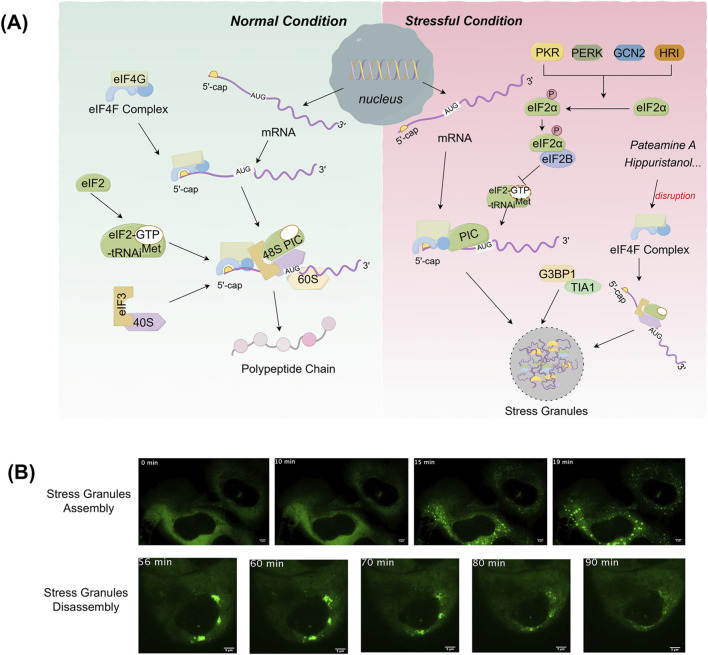
Processes of stress granules assembly. **(A)** In normal conditions, translation initiation complexes (eIF4F, eIF3, eIF2) assemble on the mRNA’s 5′ cap, recruiting the 40S and 60S ribosomal subunit. This ribosome scans the mRNA to find the start codon (AUG), moves along to synthesize the polypeptide chain. In stressful conditions, stress-activated kinases phosphorylate eIF2α, suppressing translation initiation. This triggers 48S complex aggregation that bind RBPs via liquid-liquid phase separation (LLPS) to form SGs. SGs assembly can also be triggered through alternative pathways such as eIF4F disruption without eIF2α phosphorylation. **(B)** The images were obtained from the article “Wheeler et al. (2016), Distinct stages in stress granule assembly and disassembly. Elife 5, e18413”. SGs were induced with 0.5 mM sodium arsenite (NaAsO_2_) in U-2 OS cells expressing GFP-G3BP1. The dynamics of SGs assembly and disassembly were then monitored live by fluorescence fluorescence-based images. The green fluorescent signal in the cells represents SGs. (The images reprinted with permission, Copyright Wheeler et al.).

The assembly of SGs is closely related to the regulation of mRNA translation (Figure 1). Under stress conditions, translation initiation is inhibited, resulting in the aggregation of untranslated mRNA, translation initiation factors and RNA-binding proteins (RBPs), which collectively form SGs (Youn et al., 2018). This process contrasts with that of normal mRNA translation. Under normal conditions, translation initiation complexes (including eIF4F complex, eIF3, eIF2, etc.) assemble on the 5′ cap structure of the mRNA, recruiting the 40S ribosomal subunit. The ribosome then scans the mRNA to locate the initiation codon (AUG), moves along the mRNA to synthesize the polypeptide chain and encounters the termination codon, releasing the polypeptide chain (Sonenberg and Hinnebusch, 2009). Conversely, in stressful conditions, protein kinases such as protein kinase R (PKR), protein kinase R-like endoplasmic reticulum kinase (PERK), general control nonderepressible 2 (GCN2), heme-regulated inhibitor (HRI) are activated in eukaryotic cells. These kinases phosphorylate serine 51 of eIF2α. Phosphorylated eIF2α binds to eIF2B, inhibiting GDP–GTP exchange and decreasing the availability of the eIF2-GTP-tRNAi^Met^ ternary complex, thereby suppressing translation initiation. This translational arrest results in ribosomal disassembly and the aggregation of 48S pre-initiation complexes (PICs), which serve as the nucleation cores for SGs assembly. These complexes then binds to RBPs to form the shell of SGs (Hofmann et al., 2021). The low complexity domains (LCDs) of RNA-binding proteins mediate cross-linking and aggregation of protein-protein, protein-RNA or RNA-RNA through liquid-liquid phase separation (LLPS), which drives the assembly of SGs (Wang J. et al., 2022). In addition to eIF2α-dependent pathways, SGs assembly can also be triggered through eIF2α-independent mechanisms. For example, compounds such as pateamine A, hippuristanol and sodium selenite have been shown to inhibit translation initiation and induce SGs formation without requiring eIF2α phosphorylation (Dang et al., 2006; Anderson and Kedersha, 2009). The core components of SGs are mRNPs complexes, which include untranslated mRNAs, 40S ribosomal subunits, and translation initiation factors (Kedersha et al., 2002; Markmiller et al., 2018). Beyond these, SGs also contains a variety of proteins and signaling molecules, with their specific composition varying depending on the cell type and duration of stress (Anderson and Kedersha, 2009). Notably, non-coding RNAs have also been identified in SGs through purifying SGs cores from mammalian cells and sequencing the enriched RNAs, revealing a diverse population of ncRNAs associated with SGs (Chen et al., 2023). Additionally, G3BP1 is a key regulatory protein in SGs assembly (Wheeler et al., 2016), interacting with Caprin1, USP10 and others to form a network of protein interactions that regulate LLPS and SGs assembly (Yang et al., 2020).

The process of SGs disassembly is a multi-step, ordered and regulated by various factors. The decomposition of large SGs involves both disassembly and removal (Wheeler et al., 2016). SGs disassembly begins with the dissipation of the shell, where larger granules are first broken down into smaller aggregates, followed by disassembly or removal of the smaller aggregates (Wheeler et al., 2016). Under normal physiological conditions, once the stressors that lead to SGs assembly are eliminated, the cell initiates the depolymerization process of SGs to restore normal cellular functions, such as resuming the translation of suspended mRNA (Wang B. et al., 2019). G3BP1 ubiquitination directly drives the depolymerization of SGs. The depolymerized components are then recognized and degraded by the autophagy system, which accelerates cell recovery after stress. Ubiquitination of G3BP1 is a key signal driving SGs disassembly, revealing a new pathway for SGs homeostasis regulation (Yang et al., 2023). Heat shock induces K63-linked polyubiquitination of G3BP1 (Hornbeck et al., 2015), with the major modification sites located at lysine residues K36, K50, K59, and K64 of the NTF2L structural domain. In contrast, other stresses, such as oxidative or osmotic stress, do not induce G3BP1 ubiquitination. Ubiquitinated G3BP1 binds the endoplasmic reticulum membrane protein FAF2 via the N-terminal NTF2L domain, recruiting VCP/p97 (Maxwell et al., 2021), which extracts G3BP1 from SGs and reduces the “osmotic threshold” of the intragranular interaction network, thereby triggering SGs disassembly. Additionally, SGs induced by short-term stress (<60 min) depolymerize though an autophagy-independent pathway, relying on G3BP1 ubiquitination and VCP/FAF2-mediated dynamic disassembly (Wang B. et al., 2019). However, under long-term stress (>90 min), SGs switch to an autophagy-dependent degradation pathway, which requires the involvement of VCP and lysosomes (Buchan et al., 2013). Studies have shown that the disassembly mechanisms of SGs induced by different stressors are different. For example, arsenite dependence ZFAND1 recruits p97 and the 26S proteasome to clear SGs by degrading abnormal components (Turakhiya et al., 2018), which further reveals the complex regulatory mechanism of the depolymerization process of SGs.

## 3 Biological role of SGs

SGs have been shown to exhibit complex and sometimes contradictory roles in the progression of various diseases, including viral infections, neurodegenerative disorders, and cancers. On one hand, SGs can form a protective barrier that helps cells respond to acute stress and maintain homeostasis. On the other hand, dysregulation of SG dynamics—such as aberrant assembly, disassembly, or compositional alterations—can disrupt key cellular processes such as autophagy and inflammatory response, thereby accelerating pathological damage and tissue degeneration.

The role of SGs in cellular responses to viral infections is complex and multifaceted (Bonenfant et al., 2019). From the perspective of cellular defense, the formation of SGs may serve as a protective mechanism by which host cells respond to viral invasion, thereby limiting viral propagation. SGs can inhibit viral proliferation by sequestering viral RNA and associated proteins, thus impairing the translation and replication of viral genomes. In certain contexts, SGs formation helps prevent viral spread and protect cells from further viral assault. Conversely, various viruses manipulate SGs assembly to counteract the antiviral response of the host cell. Viruses such as influenza, human immunodeficiency virus-1 (HIV-1), rotaviruses, and novel coronaviruses are known to interfere with SGs assembly to evade host defense mechanisms (Malinowska et al., 2016; McCormick and Khaperskyy, 2017). In the case of coronavirus infections, although SGs-associated proteins co-localize with polypyrimidine tract-binding proteins (PTBs), this co-localization does not necessarily indicate an antiviral role of SGs. PTBs have been shown to negatively regulate viral RNA accumulation, and suppression of PTB expression leads to increased levels of viral mRNA and enhanced virulence (Zúñiga et al., 2004). This suggests that viruses may exploit SGs by interacting with host proteins such as PTB to create a favorable environment for viral transcription, translation, or assembly (Sola et al., 2011). Moreover, studies have shown that the novel coronavirus N protein binds to G3BP, inhibiting the formation of G3BP-regulated SGs, thereby attenuating host antiviral innate immunity to facilitate SARS-CoV-2 replication *in vivo* (Liu H. et al., 2022; Zheng et al., 2022). Therefore, targeting specific host proteins involved in SGs assembly or in the interaction between SGs and viral components may offer a promising therapeutic strategy against viral infections (Mahboubi and Stochaj, 2017).

While SGs exert protective effects under physiological conditions, they can contribute to the development of neurodegenerative diseases under pathological conditions (Protter and Parker, 2016). Under normal conditions, once external stimuli such as heat shock or oxidative stress are removed, SGs are rapidly disassembled, allowing mRNA to re-engage in translation and thereby restoring cellular metabolic homeostasis (Wolozin and Ivanov, 2019). However, during the progression of neurodegenerative diseases, the clearance of SGs becomes impaired. Studies have shown that in amyotrophic lateral sclerosis (ALS), frontotemporal dementia (FTD) and Alzheimer’s disease (AD), persistent chronic stress leads to sustained SGs assembly, with many disease-associated RNA-binding proteins abnormally accumulating within SGs (Bosco et al., 2010; Dormann and Haass, 2011). These proteins may misfold and aggregate into insoluble amyloid fibrils, ultimately impairing neuronal function and promoting cell death, thus accelerating neurological disease progression. Given the pivotal role of SGs in the pathology of neurodegenerative disorders, SGs-related pathways represent promising therapeutic targets (Becker et al., 2017). Strategies aimed at reducing the aggregation of pathogenic proteins—either by inhibiting SGs assembly or enhancing their disassembly—may help mitigate disease progression. Notably, small molecule inhibitors targeting SGs-related signaling pathways, such as compounds that inhibit the eIF4 pathway and eIF2α pathways, have demonstrated neuroprotective effects in experimental models of neurodegenerative diseases (Smith and Mallucci, 2016).

During tumor evolution, various stressors, such as hypoxia, oxidative stress, endoplasmic reticulum stress, nutrient deprivation and DNA damage, not only accompany cancer progression (Solimini et al., 2007; Seebacher et al., 2021), but also serve as driving forces for tumor growth, metastasis, and therapeutic resistance (Hayes et al., 2020; Akman et al., 2021). These stressors can act as hormetic stimuli, wherein low doses induce adaptive cellular responses that enhance survival, while high doses result in cytotoxicity—a phenomenon referred to as hormesis (Mattson, 2008). In the tumor microenvironment, such hormetic stress promotes adaptive changes that confer survival advantages to cancer cells, in which SGs assembly and disassembly play a pivotal regulatory role (Hung et al., 2003; Zhang et al., 2011). In normal cells, failure to resolve SGs under prolonged stress conditions can activate apoptotic signaling and lead to programmed cell death. However, cancer cells appear to evade this fate by efficiently clearing SGs, thereby suppressing apoptosis, maintaining intracellular homeostasis, and ensuring their own survival and proliferation. This capacity to modulate SGs dynamics is considered a critical mechanism by which cancer cells sustain growth under adverse conditions (Wang A. et al., 2022). Studies have demonstrated that SGs assembly is detectable in various tumors, including pancreatic cancer, lung cancer, colon cancer, prolactinoma (Dolicka et al., 2021; Libert et al., 2024), and is strongly associated with cancer cell drug resistance. In KRAS-mutated pancreatic, lung, and colon cancer cells, the number of SGs significantly increases upon exposure to chemotherapeutic agents or radiation. These tumor cells further promote SGs assembly though the secretion estrogen-like molecules (15-day-PGJ2), thereby enhancing resistance to chemotherapy (Grabocka and Bar-Sagi, 2016). Similarly, in prolactinomas, aberrant cholesterol metabolism has been found to promote SG assembly and impair membrane localization of the dopamine D2 receptor (DRD2), resulting in resistance to dopamine agonists such as cabergoline (Peng et al., 2025).

**FIGURE 2:**
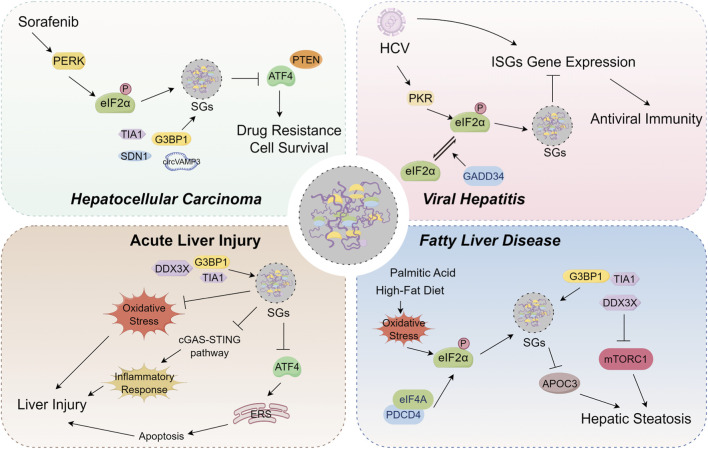
Overview of the pathophysiological roles of stress granules (SGs) on hepatocellular carcinoma, viral hepatitis, acute liver injury and fatty liver disease. In hepatocellular carcinoma, SGs are composed of specific components, including SDN1, TIA1, and CircVAMP3. Sorafenib can induce SGs assembly by activating the PERK pathway and promoting eIF2α phosphorylation. This Sorafenib-induced SGs formation limits ATF4 expression, thereby promoting cancer cell survival and conferring drug resistance. Furthermore, the formation of SGs also contributes to tumor progression and sorafenib resistance by inhibiting the translation of the tumor suppressor PTEN. In viral hepatitis, HCV infection induces PKR-dependent eIF2α phosphorylation and SGs assembly, which downregulates the translation of antiviral interferon-stimulated genes (ISGs) in hepatocytes. This process blocks the antiviral effects of interferon and promotes persistent viral infection. Separately, GADD34 contributes to SGs disassembly by facilitating the dephosphorylation of eIF2α. This promotes dynamic SGs oscillations that prevent long-term translational arrest and enable the maintenance of persistent infection. In acute liver injury, the core SGs proteins DDX3X, G3BP1, and TIA1 participate in SGs assembly. Subsequently, these SGs regulate the progression of liver injury by modulating oxidative stress, the cGAS-STING pathway, and ATF4-modulated apoptosis. In fatty liver disease, palmitic acid or high-fat diet drives SGs assembly by inducing eIF2α phosphorylation. These SGs or SGs core protein DDX3X can subsequently modulate hepatic steatosis by regulating the expression of apolipoprotein C3 (APOC3) and the mTORC1 signaling pathway.

Accumulated evidence indicates that SGs are widely involved in the progression of various liver diseases, and that their dynamic assembly and disassembly are closely associated with disease pathogenesis. In this context, the present review comprehensively examines the mechanisms underlying SGs assembly, as well as their pathophysiological roles across different categories of liver diseases (Figure 2), with the aim of providing a comprehensive reference for future research.

## 4 Biological role of SGs in liver disease

### 4.1 Liver cancer

Primary liver cancer mainly comprises hepatocellular carcinoma (HCC) and intrahepatic cholangiocarcinoma, along with rare subtypes such as hepatic angiosarcoma and hepatoblastoma (Liang et al., 2022). HCC is the most common histological type of primary liver cancer and represents a major global health burden. In 2018, it ranked as the seventh most commonly diagnosed cancer worldwide and the second leading cause of cancer-related mortality (Kulik and El-Serag, 2019; McGlynn et al., 2020). Epidemiologic studies have identified that the major risk factors for liver cancer are chronic hepatitis B virus (HBV) or hepatitis C virus (HCV) infection, consumption of aflatoxin-contaminated food, excessive alcohol intake, obesity, type 2 diabetes mellitus and tobacco use (Cao et al., 2021). Globally, the incidence of HCC is positively correlated with age, with the peak incidence occurring around the age of 75 years (Petrick et al., 2020), and the incidence of primary HCC in males is 2–4 times higher than that in females (Sung et al., 2021). Older age, male sex, and asian are considered significant risk factors for liver cancer (Zhang et al., 2022). There are significant geographic differences in risk factors for liver cancer in different regions of the world, which are closely related to factors such as environmental exposures, lifestyles patterns, infectious disease prevalence, and genetic backgrounds (Lafaro et al., 2015). In China, where liver cancer is highly prevalent, the major risk factors include chronic HBV infection and aflatoxin exposure (Lin et al., 2023), whereas in other countries such as Japan, Italy, and Egypt, HCV infection is the primary cause of liver cancer (Sung et al., 2021).

The prognosis of HCC is relatively favorable if diagnosed and treated at an early stage. However, due to its asymptomatic nature, most cases are detected at an intermediate or advanced stage, resulting in poor outcomes (Siegel et al., 2022). Currently, the most effective treatment options for HCC remains surgical resection or liver transplantation (Lo, 2013). Nevertheless, not all patients are eligible for surgery, and the 5-year recurrence rate after surgical treatment approaches 70% (Santopaolo et al., 2019; Ren et al., 2024). Moreover, due to the high malignancy and aggressiveness of HCC, many patients are not suitable candidates for surgical resection and instead depend on radiotherapy, chemotherapy, targeted therapy, and immunotherapy. Unfortunately, tumor cell resistance often leads to significantly reduced efficacy of chemotherapy and targeted therapy in HCC patients (Zhou et al., 2022).

Emerging evidence indicates that SGs assembly can be detected in many types of cancer cells, and that approximately one-third of the transcribed substances and proteins in SGs are functionally involved in classical cancer-related processes (Wang R. et al., 2019). However, the oncogenic role of SGs has not been clearly elucidated, and current findings only suggest that SGs may play an important role in carcinogenesis (Dolicka et al., 2021). Notably, even in the absence of cellular stress, overexpression of specific factors involved in the SGs assembly is sufficient to trigger the assembly of SGs, whereas suppression of their expression or activity significantly impedes SGs assembly under stress conditions. Dysregulation of these SGs-related components is frequently observed in HCC through various mechanisms, and experimental evidence suggests that they may be involved in HCC onset and progression (Anderson et al., 2015; Dolicka et al., 2021). Nonetheless, direct evidence delineating the precise role of SGs assembly in HCC development and progression remains lacking. Several studies using bioinformatics analyses have identified a range of SGs-related genes that are aberrantly expressed in HCC. For instance, a study based on the Cancer Genome Atlas-Liver Hepatocellular Carcinoma (TCGA-LIHC), GSE25097 and GSE36376 datasets identified key prognostic genes by screening with the Least absolute shrinkage and selection operator (LASSO) model to construct risk scores and performing multifactorial Cox regression analysis. Seven SGs-related genes—DDX1, DKC1, BICC1, HNRNPUL1, CNOT6, DYRK3 and CCDC124—were found to be differentially expressed in HCC and significantly associated with patient prognosis. Gene Ontology (GO) and Gene Set Enrichment Analysis (GSEA) indicated that these SGs genes might be involved in biological processes such as the cell cycle and RNA editing (Ren et al., 2024). Another study developed a prognostic signature based on four SGs-related genes (KPNA2, MEX3A, WDR62 and SFN) by bioinformatics analysis, demonstrating the potential of SGs-related markers in predicting HCC prognosis (Li M. et al., 2024). In terms of SGs-associated RNAs, sequencing of RNAs from purified SGs in SMMC-7721 HCC cells revealed the expression characteristics of both linear RNA and circular RNAs (circRNAs). CircRNAs with strong binding affinity to SGs-associated RBP were more likely to be enriched in SGs. Among them, circEXOC6B, circCALD1 and circVAMP3 were significantly downregulated in HCC tissues compared to adjacent normal tissues, suggesting a potential regulatory role of circRNAs in SGs assembly and HCC progression (Chen et al., 2023). Collectively, these findings demonstrate that SGs-related genes are aberrantly expressed in HCC and are closely associated with patient prognosis and tumor behavior, thereby offing novel insights and potential targets for diagnosis and therapy.

As protective cellular structures formed under stress, SGs facilitate the survival, proliferation, and migration of HCC cells. As a one of core RNA-binding proteins involved in SGs assembly, studies have shown that TIA1 deficiency aggravates hepatic lipid deposition and exhibits a dual role in HCC development. On the one hand, TIA1 functions as a tumor suppressor during early stages of tumorigenesis, with downregulated in human HCC tissues. On the other hand, TIA1 has been found to promote proliferation, migration, and invasion of HCC cells (Dolicka et al., 2022), highlighting the complex and context-dependent roles of SGs in HCC. In HCC cell lines such as Huh-7 and HepG2, SGs assembly contributes to the cellular stress response by mitigating or delaying stress-induced damage and promoting cancer cell survival, partly through the reprogramming of intracellular energy metabolism (Salleron et al., 2014; Li M. et al., 2023). Moreover, studies have been reported to sequester caspase-3/7 through evolutionarily conserved residues in their catalytic domains, thereby suppressing stress-induced apoptosis and supporting tumor progression under pathological conditions (Fujikawa et al., 2023). SGs also regulate the expression of cell cycle-related proteins that affect HCC cell proliferation and migration. For example, CircVAMP3, which is abundantly expressed in normal tissues but significantly downregulated in HCC, exemplifies this mechanism. Its expression negatively correlates with tumor size, and functional assays revealed that circVAMP3 binds the RGG domain of CAPRIN1, forming a CAPRIN1-G3BP1 complex that drives CAPRIN1 phase separation and promotes SGs assembly. Moreover, circVAMP3 can bind to c-Myc mRNA and inhibit its translation via SGs, thereby suppressing HCC cell proliferation, growth, and metastasis (Chen et al., 2023). SGs-related genes (KPNA2, WDR62, MEX3A, and SFN), which are highly expressed in human HCC tissues, promote HCC development via G3BP1-dependent SGs assembly. These SGs serve as a protective mechanism against hostile microenvironments, such as hypoxia, acidity, and oxidative stress. Silencing any of these four genes significantly reduces the metastatic potential of HCC cells (Li M. et al., 2024).

SGs play an important role in the resistance of HCC to chemotherapy and targeted therapy. The atypical kinase RIOK1 has been shown to mediate the sequestration of PTEN mRNA into SGs via liquid–liquid phase separation (LLPS), suppressing PTEN translation and contributing to tumor progression and resistance to tyrosine kinase inhibitors (TKIs) (Meng et al., 2025). Additionally, Sorafenib, the primary targeted agent used for advanced HCC, often encounters resistance. Studies have shown that sorafenib induces SGs assembly in various cancer cell types, including those from breast, prostate, and cervical origins (Asadi et al., 2021). Among them, in sorafenib-sensitive Hep3B cells, SGs are formed via eIF2α phosphorylation mediated by PERK activation, which reduces translation and limits ATF4 expression by sequestering its mRNA from polysomes (Adjibade et al., 2015). Low ATF4 levels support cell survival by promoting antioxidant and chaperone gene expression (Han et al., 2013). In contrast, weak SGs assembly in Huh-7 cells fails to sequester ATF4 mRNA, leading to excessive translation and pro-apoptotic signaling (Adjibade et al., 2015). Further investigations revealed that the RNA demethylases ALKBH5 and FTO modulate ATF4 expression by removing N6-methyladenosine (m^6^A) modifications at a conserved 5′UTR site (A235), enabling ribosomes to bypass upstream open reading frames (uORFs) and initiate ATF4 translation. This regulation operates independently of SGs sequestration, allowing precise control of ATF4 expression and balancing cell survival and death under stress (Adjibade et al., 2024). Mutant p53 (mutp53) have been associated with improved survival in HCC patients compared to those with p53-null tumors. Mechanistically, mutp53 inhibits SGs assembly by binding PERK and suppressing eIF2α phosphorylation, as well as by binding G3BP1 and blocking its oligomerization. This enhances sensitivity to endoplasmic reticulum stress and promotes sorafenib-induced apoptosis (Thoenen et al., 2025). Hence, targeting SGs assembly may enhance HCC sensitivity to sorafenib. The catalytic subunit of PI3K, p110α, promotes SGs assembly via its Arg-Gly (RG) motif in HepG2 and Huh7 cell lines. Methylation of the RG motif of p110α by protein arginine methyltransferase 1 (PRMT1) inhibits SGs assembly and reduces sorafenib resistance. Based on this mechanism, the metal-polyphenol network-coated R612F nanoparticles (MPN-R612F) were developed to maintain the hypermethylated state of p110α in HCC cells, which inhibits SGs assembly. This ultimately reduce the resistance of HCC cells to sorafenib, thereby enhancing its antitumor efficacy (Zhou et al., 2024).

Although no direct studies have examined the specific role of SGs in chemotherapy response in HCC, available evidence supports their involvement in chemoresistance. Staphylococcal nuclease and tudor domain containing 1(SND1), as an SGs-specific protein, dynamically regulates SGs assembly under heat shock and oxidative stress (Gao et al., 2010; Gao et al., 2015). SND1 is overexpressed in HCC and exerts anti-apoptotic effects by modulating the expression of lncRNA UCA1, thereby reducing sensitivity to 5-fluorouracil (5-FU)-induced apoptosis (Cui et al., 2018). In cisplatin-treated HCC cells, CD147, as a tumor-associated membrane protein, promotes SGs disassembly by binding G3BP1 and facilitating its transport to lysosomes via Rab7A. This suppresses mTORC1 signaling, induces cytoprotective autophagy, and decreases chemosensitivity (Qian et al., 2024).

Although numerous studies have highlighted the close relationship between SGs formation and the onset, proliferation, migration, and chemotherapy resistance of HCC, direct evidence elucidating the specific role of SGs in the pathogenesis and progression of HCC or other type of liver cancer remains limited. Much of the current research relies on HCC cell lines or animal models, while clinical validation and studies using human samples are still insufficient. Therefore, future studies must include more clinical data to confirm the actual role and clinical significance of SGs in HCC patients. Moreover, although SGs are implicated in chemotherapy resistance, most existing studies focus primarily on resistance mechanisms related to specific drugs, such as sorafenib. There is still a notable lack of research on SGs regulation strategies targeting various drug resistance mechanisms across different liver cancer subtypes.

### 4.2 Viral hepatitis

Viral hepatitis refers to a group of liver inflammatory diseases caused by various viruses. Based on the specific pathogen, viral hepatitis can be classified into several types, including hepatitis A, B, C, D and E. Viral hepatitis presents a significant global public health challenge, with its epidemiological status having a profound impact on societal health. Among these, hepatitis B virus (HBV) and hepatitis C virus (HCV) infections are leading causes of hepatitis and related liver diseases (Schillie et al., 2018; Sayiner et al., 2019). HCV is a single-stranded, positive-stranded RNA virus that can lead to cirrhosis and liver cancer upon infection. Globally, more than 184 million people are chronically infected with HCV (Thrift et al., 2017). And, there are significant geographic variations in the incidence and prevalence of HCV (Zhao et al., 2012). Moreover, a large proportion of HCV infections go undetected, and untreated HCV can lead to severe hepatic complications, including cirrhosis, hepatic failure, and HCC, resulting in up to 500,000 related deaths annually (Guirao et al., 2006).

HCV infection is particularly associated with SGs assembly in hepatocytes. As an RNA virus, HCV encodes a single polyprotein whose translation is driven by an internal ribosome entry site (IRES) in the 5′ untranslated region (5′UTR). Although HCV infection causes the expression of antiviral interferon-stimulating genes (ISGs) in the liver, HCV is able to persist by inhibiting the effector functions of these ISGs. Research indicates that HCV infection triggers the phosphorylation of PKR and eIF2α, leading to a reduction in cellular protein synthesis. Additionally, HCV infection induces the formation of PKR-dependent SGs, downregulating the translation of intracellular antiviral genes. Interferon further promotes SGs assembly by enhancing PKR phosphorylation, thus blocking protein translation of ISGs despite high mRNA expression (Garaigorta and Chisari, 2009; Garaigorta et al., 2012). HCV infection inhibits ISG protein expression by phosphorylating PKR, thereby blocking the antiviral effects of interferon and promoting persistent viral infection. Inhibition of PKR expression restores the antiviral effects of interferon in infected cells (Garaigorta and Chisari, 2009; Dabo and Meurs, 2012). Cyclosporine, a widely used immunosuppressant for the prevention of rejection after organ transplantation (Winkler, 2000), also functions as a modulator of IFN signaling pathway at the translational level. Studies have shown that the cyclosporine inhibitor SCY-635 disrupts the interaction between cyclosporine and PKR, reduces the phosphorylation of PKR and eIF2α, and inhibits SGs assembly in HCV-infected cells, thereby restoring the ISGs expression and enhancing the anti-HCV activity of cyclosporine A (Daito et al., 2014). It has been demonstrated that the translation initiation of HCV IRES in human cells is independent of translation initiation factors such as eIF2, eIF2α, eIF4α (González-Almela et al., 2018), meaning that HCV translation and replication are not significantly impacted by SGs formed through the PKR-eIF2α axis. Moreover, the presence of SGs in HCV-infected cells was negatively correlated with the expression of ISGs (e.g., MxA and USP18), suggesting that SGs may inhibit the translation of antiviral genes and provide conditions for viral escape immunization. In contrast, HCV may escape the mRNA translation blockade imposed by SGs through Stau1, a double-stranded RNA-binding protein involved in the regulation of mRNA transport, localization, and translation. Studies have shown that Stau1 binds to the IRES structural domains of both the 3′NTR and 5′NTR of HCV, promoting viral replication and translation. Additionally, Stau1 inhibits PKR autophosphorylation and thus prevents PKR-mediated inhibition of eIF2α, which is conducive to HCV protein synthesis and efficient translation and replication of viral RNA (Dixit et al., 2016). It was also found that the SGs proteins TIA-1, TIAR, and G3BP1 are essential for the accumulation of HCV RNA and protein and play a critical role in viral assembly and release (Garaigorta et al., 2012). Ming-Chih Lai et al. also found that HCV can evade immune system surveillance by affecting DDX3, an RNA helicase involved in SG formation. DDX3 promotes the translation of PACT mRNAs, which are involved in the recognition of viral RNAs, but HCV core proteins sequester DDX3 into SGs, inhibiting PACT translation (Lai et al., 2016). These findings highlight two mechanisms by which HCV exploits SGs: first, by inducing SGs assembly through PKR phosphorylation, which inhibits the translation of antiviral genes, and second, by using SGs proteins to promote viral replication and release.

Additionally, it has been reported that HCV can regulate viral gene expression through the regulation of cytoplasmic processing bodies (P-bodies) and SGs (Pager et al., 2013). P-bodies are dynamic, membrane-encapsulated subcellular structures present in the eukaryotic cytoplasm that are involved in mRNA degradation, storage, and translational repression (Eulalio et al., 2007; Luo et al., 2018). SGs and P-bodies work together to determine mRNA fate: SGs offer temporary storage and protection, while P-bodies mediate silencing and degradation. This coordinated interaction enables cells to suppress non-essential protein synthesis during stress while conserving crucial genetic information for recovery. During stress conditions, translation initiation is inhibited, and untranslated mRNAs are sequestered into SGs to avoid mistranslation or degradation. After stress relief, mRNAs may either re-enter translation or be directed to P-bodies for degradation, forming an integrated mRNA metabolic network. HCV infection can specifically inhibit P-body formation in hepatocytes, disrupting mRNA regulation and potentially promoting aberrant gene expression in hepatocytes thereby triggering HCV-associated liver disease (Perez-Vilaro et al., 2015). Concurrently, HCV infection promotes SGs assembly, and immunofluorescence studies have shown that many P-body and SGs proteins are co-localized with the viral core proteins in the lipid droplets (Pager et al., 2013), which are the HCV viral RNA packaging site. Previous research has indicated that HCV activates IKK-α through interaction with DDX3X, which in turn induces a transcriptional program linked to lipogenesis. This enhances the binding of the viral core to lipid droplets and promotes viral assembly (Li et al., 2013; Pène et al., 2015; Lowey et al., 2019). Reducing P-bodies and SGs proteins diminishes HCV RNA and viral particle numbers, yet reducing SGs proteins instead enhanced the accumulation of infectious viral particles. These findings suggest that HCV hijacks P bodies and SGs components to facilitate viral gene expression and viral replication in specific cytoplasmic locales (Ariumi et al., 2011; Pager et al., 2013; Fernandez-Carrillo et al., 2018).

Persistent viral infection is closely associated with the phenomenon of SGs oscillations. In HCV-infected Huh7 cells, the combined effects of HCV infection and type I interferon (IFN-α) leads to a highly dynamic SGs assembly and disassembly accompanied by active and stalled translational phases, delayed cytokinesis, and prolonged cell survival. These oscillations are driven by PKR-mediated eIF2α phosphorylation, which is counteracted by GADD34 dephosphorylation. Double-stranded RNA (dsRNA) formed during HCV infection activates PKR/eIF2α to initiate SGs assembly, whereas GADD34 contributes to SGs disassembly by facilitating the dephosphorylation of eIF2α. This regulation creates a negative feedback loop, promoting dynamic SGs oscillations that prevent long-term translational arrest and enable the maintenance of persistent infection (Ruggieri et al., 2012). SGs oscillations represent a universal mechanism to prevent prolonged translational inhibition and are a conserved host response to RNA viruses, potentially allowing HCV to establish chronic infection.

In conclusion, the role of SGs in HCV hepatitis studies exhibits multidimensional regulatory features. SGs are both executors of host defenses and targets of exploitation for viral evolution, and involved in broad translational regulation and local reorganization of viral replication complexes. HCV optimizes replicative environments by modulating SGs assembly and exploiting host RNA-binding proteins to ensure persistent replication. While current studies on SGs in viral hepatitis have predominantly focused on HCV, less is known about the role of SGs in other hepatitis viruses. Notably, it has been reported that the RNA helicase DDX3 binds to the HBV viral polymerase (Pol), merges into viral particles, and inhibits their reverse transcription (Ariumi, 2014; Valiente-Echeverría et al., 2015). Despite being a DNA virus, HBV replicates its DNA through reverse transcription of its genome, which also suggests that SGs may be involved in the HBV infection process. However, it remains unclear whether SGs are formed during HBV infection and how they impact the HBV replication cycle. Furthermore, studies on the involvement of SGs in hepatitis A virus (HAV), hepatitis D virus (HDV), and hepatitis E virus (HEV) infections are limited. It is worth noting that HAV, HDV and HEV are all RNA viruses, so their potential effects on SGs may have some similarities with HCV, but this hypothesis still needs to be further validated and elucidated in future studies.

### 4.3 Acute liver injury

Acute liver injury (ALI) is a clinical syndrome characterized by acute onset and rapid progression, posing a serious threat to human health (Lee et al., 2008). Without timely diagnosis and treatment, ALI may rapidly progress to acute liver failure (ALF), leading to multiple organ failure with a mortality rate exceeding 35% (Jin et al., 2021). The etiology of ALI is complex and multifactorial, including drugs or toxins, viral hepatitis infections, immune-mediated factors, ischemic injury, metabolic disorders, bacterial infections, trauma, and surgical complications. The causative factors and incidence rates of ALI vary geographically, with viral hepatitis being the predominant cause in Africa, India, and other Asia-Pacific regions (European Association for the Study of the Liver et al., 2017), while drug-induced liver injury (DILI) is more commonly observed in Western countries due to drug overuse. DILI is one of the most common adverse reactions to clinical medications and represents the most frequent type of ALI, which may progress to acute liver failure or even result in death in severe cases (Björnsson and Björnsson, 2022). In Western countries, acetaminophen (APAP) overdose is the primary cause of DILI (Cui et al., 2018). In China, the annual incidence of DILI has reached 23.8 cases per 100,000 individuals, surpassing that observed in Western nations. Epidemiological studies indicate that the inappropriate use of herbal medicines, dietary supplements, and antituberculosis drugs are the main contributors to DILI in mainland China (Shen et al., 2019).

The pathogenesis of ALI is multifaceted, involving various pathological processes, including oxidative stress, inflammatory responses, metabolic disturbances, mitochondrial dysfunction, and hepatocyte necrosis and apoptosis. Recent studies have highlighted the involvement of SGs assembly in the progression of ALI. For instance, intraperitoneal injection of 10 mg/kg sodium arsenite has been shown to induce SGs assembly in liver tissue, and this process is regulated by circadian rhythms mediated by the phosphorylation and dephosphorylation of oscillatory eIF2α (Wang R. et al., 2019). DDX3X, a key regulator of SGs assembly, modulates SGs assembly in the liver and protects against ALI induced by APAP, carbon tetrachloride (CCl_4_), and thioacetamide (TAA). Treatment with APAP in primary hepatocytes isolated from DDX3X^fl/fl^ or DDX3X^Δhep^ mice induce SGs assembly in hepatocytes. Mechanistic studies have suggest that DDX3X confers protection by regulating SGs formation and oxidative stress, with hepatocytes deficient in DDX3X being more susceptible to oxidative stress and inflammation, resulting in more severe liver injury (Luo et al., 2023). Furthermore, human bone marrow mesenchymal stem cells (hMSCs) in APAP-induced ALI have been shown to promote the expression of SGs marker proteins G3BP1 and TIA-1 in hepatic tissue and induce M2 macrophage polarization, thus reducing liver injury. *In vitro* co-culture experiments with RAW264.7 cells and hMSCs demonstrated that hMSCs can inhibit cGAS production in hepatocytes by inducing M2 macrophages to produce SGs, suppressing the cGAS-STING pathway and attenuating the inflammatory response (Wang et al., 2024). In hypoxia-induced ALF, prolonged hypoxia exacerbates cellular injury, endoplasmic reticulum stress, and apoptosis. However, SGs levels, marked by G3BP1 and TIA-1 expression, initially increase and subsequently decrease, suggesting that early SGs assembly may serve as a protective response, while its decline may indicate the weakening of cytoprotective mechanisms. Arsenite-induced SGs assembly can mitigate the progression of liver injury by reducing apoptosis through the modulation of ATF4-mediated endoplasmic reticulum stress (Li W.-Y. et al., 2023). Moreover, the role of G3BP1 in ALI has been increasingly recognized. Wen-Yuan Li et al. investigated the prognostic value of plasma G3BP1 levels in patients with ALF and acute-on-chronic liver failure (ACLF) undergoing artificial liver support system (ALSS) treatment. Using Cox regression and random forest modeling, they found that elevated G3BP1 expression was positively correlated with improved prognosis in liver failure patients. These findings suggest that G3BP1 may serve as a potential biomarker for assessing liver failure prognosis, providing valuable guidance for clinical interventions, including liver transplantation (Li W.-Y. et al., 2024).

In summary, current studies suggest that SGs plays a significant role in the pathological process of ALI. However, no studies have systematically investigated the molecular mechanisms underlying SGs assembly and disassembly during ALI, nor the effects on ALI progression. Furthermore, it remains unclear whether SGs assembly consistently occurs across various types of ALI induced by different etiologies, warranting further investigation. Moreover, there is a lack of clinical ALI samples to directly confirm the impact of SGs on ALI progression. Comprehensive modulation of the SGs regulatory network through molecularly targeted interventions remains in the early stages of investigation. With continued research, SGs regulation is expected to become a crucial avenue for the prevention and treatment of ALI.

### 4.4 Fatty liver disease

Fatty liver disease encompasses a spectrum of chronic liver co disorders driven by diverse etiologies. It can be classified into metabolic dysfunction-associated fatty liver disease (MAFLD), metabolic and alcohol-associated liver disease (MetALD), alcohol-associated liver disease (ALD), other etiology-specific fatty liver diseases, cryptogenic fatty liver disease, and metabolic dysfunction-associated steatohepatitis (MASH) (Rinella et al., 2024). The etiology of fatty liver disease is complex and multifactorial, including metabolic abnormalities, chronic alcohol consumption, viral hepatitis, drug-induced liver injury, inherited metabolic disorders, malnutrition, and rapid weight loss. In the Asia-Pacific region, obesity and type 2 diabetes mellitus are the predominant risk factors (Kim et al., 2021). Notably, fatty liver disease in this region exhibits unique epidemiological characteristics, such as high prevalence among lean individuals, frequent co-occurrence with viral hepatitis, and a rapidly increasing incidence driven by population aging and lifestyle changes (Kawaguchi et al., 2022). Moreover, ALD remains a major contributor to cirrhosis- and liver cancer-related mortality worldwide (Gao and Bataller, 2011). The disease typically progresses from simple steatosis to steatohepatitis, fibrosis, cirrhosis, and ultimately HCC (Thursz et al., 2019).

SGs and its associated proteins have been identified as important regulatory elements in fatty liver disease. Core proteins involved in SGs assembly, including G3BP1, TIA1, DDX3X, and PDCD4, have been reported to be associated with fatty liver disease. Recent studies have reported that treatment with 0.6 mM palmitic acid (PA) induces SGs assembly in HepG2 cells, accompanied by significant upregulation of G3BP1 and TIA1 in both obese mice and PA-treated hepatocytes. Knockdown of G3BP1 exacerbates PA-induced lipid accumulation in hepatocytes and worsens high-fat diet (HFD)-induced MAFLD. Mechanistic investigations reveal that G3BP1 attenuates MAFLD progression by negatively regulating apolipoprotein C3 (APOC3) expression, which otherwise translocates to the nucleus to activate metabolic genes, promoting hepatic lipid accumulation and metabolic dysfunction (Liu et al., 2025). Furthermore, TIA1 deficiency has been shown to promote hepatic steatosis and fibrosis in metabolic liver disease, which is associated with dysregulation of genes involved in lipid metabolism and inflammation. *In vitro*, TIA1 knockdown enhances tumor cell migration and proliferation, whereas *in vivo* mouse models suggest a tumor-suppressive function (Dolicka et al., 2022). In patients with nonalcoholic fatty liver disease (NAFLD) and in HFD-induced NAFLD mouse models, DDX3X expression is markedly reduced. Knockdown of DDX3X exacerbates HFD-induced hepatic steatosis, while further studies reveal that DDX3X ameliorates steatosis during NAFLD progression by inhibiting the mTORC1 signaling pathway (Liu P. et al., 2022). PDCD4, a tumor suppressor protein involved in translational repression through its interaction with eIF4A, also plays a critical role in SGs assembly. Both oxidized low-density lipoprotein (ox-LDL) and HFD can induce oxidative stress, leading to SGs formation accompanied by increased eIF2α phosphorylation. PDCD4 promotes SGs assembly by binding eIF4A and enhancing eIF2α phosphorylation, in part through its interaction with the RNA-binding protein RBM2. Notably, PDCD4 deficiency reduces stress-induced eIF2α phosphorylation and impairs SGs assembly, suggesting a protective role in fatty liver disease (Bai et al., 2016). Jeongmin Park et al. demonstrated that tristetraprolin (TTP), an AU-rich element (ARE)-containing mRNA-binding protein, attenuates senescence-associated fatty liver and cellular senescence by promoting PAI-1 degradation within SGs. Conversely, TTP deficiency exacerbates hepatic inflammation, senescence-associated secretory phenotype (SASP) production, and lipid accumulation. In addition, carbon monoxide (CO) activates the Sirt1-TTP axis and PERK-eIF2α signaling pathway, thereby inhibiting SGs assembly and PAI-1-mediated cellular senescence, ultimately reducing age-dependentPDCD4 (Park et al., 2023).

At present, most studies on SGs in fatty liver disease focus on MAFLD induced by HFD. However, there is a significant lack of research addressing the roles of SGs and their core components in ALD, steatohepatitis, and other etiology-specific fatty liver diseases. Additionally, there is a lack of systematic exploration of the molecular mechanisms by which SGs and their associated proteins influence the progression of fatty liver disease. Moreover, systematic exploration of the molecular mechanisms through which SGs and their associated proteins influence disease progression remains limited. Future research should aim to elucidate the specific molecular pathways and pathophysiological roles of SGs in diverse subtypes of fatty liver disease, and assess their potential as diagnostic biomarkers or therapeutic targets. Such efforts will enhance our understanding of the heterogeneous pathological mechanisms underlying fatty liver disease and support the development of precision medicine approaches.

### 4.5 Other liver diseases

In addition to common liver diseases such as HCC, hepatitis, hepatic steatosis, and liver injury, there are also emerging reports about the association of SGs with other types of liver diseases. Beyond classical viral hepatitis viruses, other viral infections may also contribute to liver diseases. The yellow fever virus (YFV), a mosquito-borne RNA virus of the *Flavivirus genus*, primarily targets the liver and can cause severe manifestations, including hemorrhagic fever, with a mortality rate of up to 50% (van Leeuwen et al., 2022). SGs has been observed in YFV-infected Huh7, HepG2, and CMMT monkey epithelial cell lines. However, inhibition of SGs assembly does not appear to affect the production of inflammatory cytokines following YFV infection, suggesting that SGs may not play an essential role in the antiviral response to YFV infection (Beauclair et al., 2020). Infection of hepatocytes with mouse hepatitis coronavirus (MHV) results in translation shutdown and mRNA degradation, while also inducing increased eIF2α phosphorylation and the formation of SGs and P-bodies (Raaben et al., 2007). Hanson et al. investigated the host stress response during P*lasmodium* infection of hepatocytes and found that during *Plasmodium berghei* infection of HepG2 hepatocytes, SGs were not spontaneously formed, and the infection did not impair the host cell’s capacity to respond to exogenous stress (Hanson and Mair, 2014). This suggests that *Plasmodium* may actively evade SGs assembly, potentially as a mechanism to avoid host immune recognition and preserve intracellular homeostasis for its replication and differentiation.

## 5 Conclusion and perspective

SGs as transient and reversible membrane-free structures formed in response to various cellular stresses, have attracted increasing attention in recent years due to their emerging roles in liver diseases. Current evidence suggests that SGs may play pivotal roles in modulating hepatocyte stress responses, apoptosis, autophagy, and inflammation processes. However, their precise mechanisms by which SGs contribute to liver disease progression remain incompletely elucidated. Although several RNA-binding proteins and translation initiation factors have been confirmed to participate in SGs assembly, the associated signaling pathways, post-translational modifications (e.g., phosphorylation, acetylation), and their spatiotemporal regulatory mechanisms in hepatocytes or other type of liver cell are still poorly understood. A deeper investigation into the molecular dynamics governing SGs assembly and disassembly, as well as their regulatory networks, will facilitate a better understanding of the functional transitions of SGs under pathological conditions. Furthermore, mRNAs enriched in SGs exhibit highly dynamic changes, however, most current studies rely on fixed-cell immunofluorescence staining, which does not accurately capture their dynamics in living systems. Although live-cell imaging techniques have enabled visualization of SGs assembly and disassembly via labeling of core SGs proteins, the dynamically tracking of mRNAs or non-coding RNAs enriched within SGs remains challenging. The development and application of advanced tools, such as multi-omics integration analysis, single-molecule imaging technologies and fluorescent biosensors, are expected to provide critical insights into the real-time behavior and functional significance of SGs-enriched RNAs. Moreover, it remains unclear whether SGs exhibit similar biological functions, such as promoting survival or inducing cell death, in acute and chronic liver diseases. How SGs interact with key signaling pathways in hepatocytes to influence disease progression, and whether SGs function similarly across different hepatocyte subtypes, are also unresolved questions requiring further exploration. Notably, recent studies have demonstrated that small-molecule inhibitors targeting G3BP proteins can effectively block SGs assembly *in vitro* (Freibaum et al., 2024). Despite this, the development of specific therapeutic strategies targeting SGs or their core components remains in its infancy. In the future, high-throughput screening and rational design of small molecules, natural products, or RNA-based therapeutics capable of modulating SGs dynamics *in vitro* and *in vivo* may offer promising avenues for precision medicine in liver diseases. In conclusion, although the study of SGs in liver disease is still at an early stage, it holds significant potential to advance both mechanistic understanding and clinical application. Future research that integrates cellular and animal models with multi-omics analyses, advanced imaging technologies, and targeted interventions will be instrumental in delineating the functional roles of SGs in liver pathophysiology, ultimately providing a theoretical foundation and therapeutic targets for the prevention and treatment of liver diseases.
